# Exploring Techniques for Vision Based Human Activity Recognition: Methods, Systems, and Evaluation

**DOI:** 10.3390/s130201635

**Published:** 2013-01-25

**Authors:** Xin Xu, Jinshan Tang, Xiaolong Zhang, Xiaoming Liu, Hong Zhang, Yimin Qiu

**Affiliations:** 1 School of Computer Science and Technology, Wuhan University of Science and Technology, NO. 947 Heping Road, Wuhan 430081, Hubei, China; 2 Hubei Province Key Laboratory of Intelligent Information Processing and Real-time Industrial System, NO. 1 Huangjiahu West Road, Wuhan 430065, Hubei, China

**Keywords:** vision surveillance, activity recognition, surveillance system, performance evaluation

## Abstract

With the wide applications of vision based intelligent systems, image and video analysis technologies have attracted the attention of researchers in the computer vision field. In image and video analysis, human activity recognition is an important research direction. By interpreting and understanding human activities, we can recognize and predict the occurrence of crimes and help the police or other agencies react immediately. In the past, a large number of papers have been published on human activity recognition in video and image sequences. In this paper, we provide a comprehensive survey of the recent development of the techniques, including methods, systems, and quantitative evaluation of the performance of human activity recognition.

## 1. Introduction

After the tragic event on September 11 and the subsequent terrorist attacks around the world, visual surveillance has attracted much more attention and has been adopted in different applications for crime detection and prediction. Automatic activity recognition is an important research direction in surveillance vision analysis. By analyzing the detected human activities, especially the abnormal activities of human beings, standoff threats can be recognized and predicted. In the past decade, a large number of in-depth research papers have been published on the recognition and understanding of human activities. They can be classified into two types of approaches: active techniques and passive techniques. Active techniques, such as radar, I/R or microwave, have been widely used to obtain images. For example, the commercial products such as the Nintendo's WII or Microsoft's Kinect are good examples that make use of active techniques [1]. However, although such products have been partially successful, their deployment per location is usually not practical in widespread public areas. Thus, we limit our work to the summarization of the past efforts on passive vision processing techniques.

**Figure 1.**
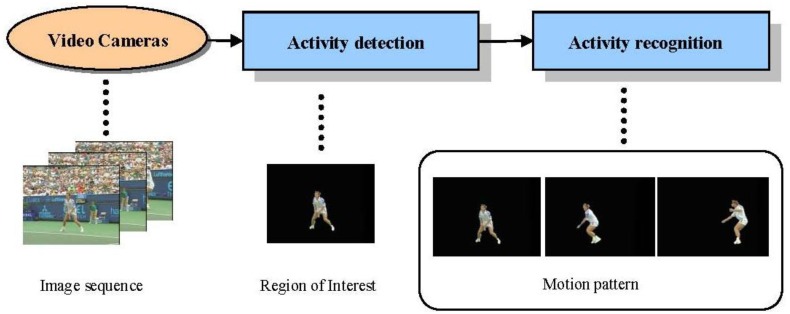
Flowchart of activity recognition.

As described in [2], activity recognition aims to draw a description of human actions and interactions through the analysis and understanding of human motion patterns. It contains two level procedures [3]. As illustrated in Figure 1, the lower level aims to detect the regions of interest (ROIs) corresponding to static or moving humans; while the higher level recognizes temporal motion patterns of human activities. From a technical viewpoint, human activity recognition can be considered as a classification problem using time varying feature data. Visual information is extracted from video sequences and represented in relevant features, which are used to match with the features extracted from a group of labeled reference sequences representing typical activities. During the extraction procedure, three kinds of features may be involved: single object's features (*i.e.*, position, velocity, veins, shape, color and *etc*.), global features of multiple objects (*i.e.*, average speed, region occupancy, relative positional variations and *etc*.), and the relationships between objects and background [4].

In the past, several efforts have been made to survey this area of research [5,6]. In [7], Popoola and Wang summarized the key points of previous related review papers on activity recognition. It is noted that previous review publications were mainly focused on the methods for building normal activity templates or normal activity models. However, these papers touch only on a subset of this research area. Our emphasis in this paper aims to discuss the existing high-level techniques, and provide summary of progress achieved in the direction of building robust and intelligent vision based methods, including abnormal activity templates, abnormal activity models, and manifold geometry. Besides, we will also discuss smart surveillance systems and evaluation metrics for human activity recognition. Beyond activity recognition, other similar fields may include event recognition, goal recognition or intent prediction. As is pointed out by [8], although these terms may emphasize different aspects of activities, their essential goals are the same. Therefore, in this paper, we use the term activity recognition and do not distinguish the minor difference between the different terms mentioned above.

The remainder of this paper is organized as follows: Section 2 discusses the methods for activity recognition. Section 3 introduces the approved surveillance systems for activity recognition. Section 4 reviews the research project on performance evaluation of activity recognition. The conclusions are given in Section 5.

## 2. Methods for Human Activity Recognition

The essence of activity recognition may be considered to be a classification problem relating to time varying data. Accordingly, two critical issues need to be addressed during classification. The first one is how to formulate the reference motion patterns for typical activities; the second one is how to enable the training and matching methods effective enough to cope with the minor deviations in both temporal and spatial scales for similar motion patterns. In different circumstances, these two problems are treated differently, and we will discuss the methods to deal with the difference from the technique viewpoint.

As stated in [9], the investigations of human activity recognition can be divided into two kinds of approaches: template matching and state space. Most previous efforts have been concentrated on using state space method to understand human activities because of its comparative high recognition accuracy [10]. Spatial features including points, lines, and blobs are used during the recognition processing. However, state space methods usually have high computational complexity while template matching methods are computationally inexpensive. Meshes of a subject image were usually applied to identify a particular movement in these methods. During the recognition processing, the features extracted from the given image sequence were compared to the pre-stored patterns. As illustrated in Table 1, we classify past research from these two directions. Typical methods are outlined below.

### 2.1. Template Matching

Template matching methods aim to extract motion features from the given image sequences, and transfer them into certain motion patterns. Then, human motion templates can be obtained from these motion patterns representing predefined activity patterns. Human normal activities can be recognized by matching the activities with the templates [24]. However, methods for normal human activity recognition may present several drawbacks when applied to anomalous activities. An anomaly can be defined as an atypical activity pattern that is not represented by sufficient samples in a training data set but critically satisfies the specificity constraint to an abnormal pattern [25]. In many applications, the data of anomalous activities is extremely scarce compared to normal activities. This may lead to significant difference in the methods for activity recognition. We will discuss the difference in this section.

#### 2.1.1. Normal Activity Template

In the early days, human activities were composed of Motion-history image (MHI) and Motion Energy Image (MEI) in different views, from which the square based motion features could be abstracted [11]. In these methods, image sequences were first processed by background subtraction and binarization. MEI can be accumulated over time by these binary motion images which contain the motion field, and enhanced to be MHI. Each activity was composed of MEI and MHI in different views, from which the square based motion features can be abstracted for template matching. However, this method can only recognize a 180 degrees angle of sample actions. Oren proposed a trainable object detection architecture that can recognize pedestrians from frontal and rear views [12]. Different from the above method, this architecture did not rely on any *a priori* model or motion template, but defined the shape of an object as a series of regions and relationships between them using wavelet templates. These wavelet templates can be used to compare with the image frames to search for the matching action.

In order to ensure human activity is invariant to viewpoint variations, Ben-Arie described these actions as temporal sequences of pose vectors that represented the motion of human body [13]. They constructed a database for major body parts, in which all the activity templates were stored in multidimensional hash tables in the form of pose entries. Voting Approach and multidimensional indexing were used in the recognition stage to improve the efficiency and stability of matching. Recently, Lu developed a system to automatically track multiple hockey players in a video sequence and simultaneously recognize their actions [14]. Hue-Saturation-Value (HSV) color histogram and Histogram of Oriented Gradients (HOG) descriptor were used to represent the color and shape information of the image of hockey players respectively. They used a 3D histogram based on the magnitude of gradients in both x and y direction and their orientations for the HOG descriptor. Thus, their method is invariant to viewpoint variations. Action templates can thus be leant and updated from training data. For a candidate action, a Sparse Multinomial Logistic Regression (SMLR) classifier can be used to classify its HOG descriptors into action categories.

#### 2.1.2. Abnormal Activity Template

Abnormal motion patterns can also be recognized through the matching of human motion templates. An internal list of anomalous motion patterns can be established as a template to match with an ongoing activity. If this ongoing activity is on the list, then it can be confirmed to be anomalous. However, this kind of approach presents several drawbacks. The significant one is that, in view of the way of generating templates, new abnormal activities cannot be discovered [15]. In order to cope with this problem, Khalid proposed a method to filter anomalous activities [15]. Instead of generating templates from motion patterns, they believed that normal behaviors possess high correlation between each other, thus abnormal activities can be detected through the comparison with normal activity recorded in video sequences. In this method, trajectories were represented as time series using modified Discrete Fourier Transform (DFT)-based coefficient in low dimensional feature space, so as to learn motion patterns using iterative hierarchical semi-agglomerative clustering-learning vector quantization. This method did not need any prior knowledge about the number and type of activity patterns. Usually, template matching methods are computationally efficient, and do not need much computation time. However, despite their low cost computation, template matching methods are sensitive to the variation of motion duration and noise, thus the accuracy of recognition is not very high.

### 2.2. State Space

Different from template matching method, the state space approach aims to formulate a statistical model through training, which can be used for the recognition of human activities [24]. In state space methods, each static posture is defined as a single state, and correlated with each other using the statistical model. Thus the motion sequence can be treated as an ergodic process through different states. For each motion sequence, the joint probability is calculated to find the maximum value [26]. State space methods can overcome the problem of motion duration variation in template matching approaches, because each state was accessed several times. However, other difficulties may arise. For example, it is far from easy to establish a fixed form model. Thus, different statistical models need to be established through complex iterative computation according to specific situation [5]. Accordingly, several graphical models were proposed to serve as an efficient way to do probability inference. Graphical model is a powerful tool for modeling dependencies among random variables, and can be divided into two categories including Directed Graphical Models (DGM) and Undirected Graphical Models (UGM) [27]. We will explore the recent efforts for generating statistical models in this section.

#### 2.2.1. Normal Activity Model

One of the most typical DGMs is the Hidden Markov Model (HMM). HMM was broadly used in speech recognition in early years, then it was successfully applied to the recognition of activities. For example, in order to model the dependence on the parameter of activity explicitly, Wilson and Bobick proposed a framework which added a global parametric variation in the output probabilities of each states in HMM [16]. In this framework, expectation-maximization (EM) method was used to train the parametric HMM. Similarly, Duong introduced the switching Hidden Semi-Markov Model (HSMM) to study and recognize human activities of daily living [17,18]. Parameters of HSMM were determined by the switching variable at the high level.

A typical example of UGM is conditional random fields (CRF), which have been emerged into behavior recognition in the last few years. Compared with HMM, CRF can easily incorporate domain knowledge and get better performance in terms of classification accuracy [28]. For example, Chieu applied CRF to solve the two behavior recognition tasks proposed at the Physiological Data Modeling Contest [19]. The Generalized Expectation Maximization was used to train the partially labeled sequences to improve the performance. Similarly, Yin proposed a dynamic conditional random field (DCRF) model based method to detect events from large-scale sensor networks in real time [20]. DCRF model incorporated temporal constraints among contiguous spatial fields, and relaxed independent spatial-temporal relationship among events in a unified probabilistic framework. Thus, it can deal with partial sensor data and interactions between contiguous events.

#### 2.2.2. Abnormal Activity Model

State space approaches set up profiles for normal activities. The activities deviating from these profiles are treated as anomalous. In other words, state space approaches construct a graphical model using a set of normal patterns to establish a classifier that can discriminate between normal and abnormal activities. The critical point of this method lies in whether or not the proposed graphical model can be used as an accurate predictor of normal activities. In this way, an ongoing pattern is likely to be anomalous when it cannot be predicted by the graphical model.

The most part of graphical models used for normal activity recognition can be also used in the detection of abnormal events. However, due to the fact that abnormal behaviors occurred rarely and were not expected in advance, these models should be adjusted according to specific applications.

Taking DGM for example, Yin and Meng proposed a self-adaptive HMM based framework to understand abnormal activities [21]. Different from the normal activities need to train from a large data set, this framework can learn on-line from current data set and generate new models for abnormal activities. In order to detect anomalies in complex outdoor scenes, Loy proposed an activity-based decomposition over complex activities, and modeled them using a cascade of DBN [22]. The activity space was factorized into sub-spaces based on exploring of the behavior semantics within the spatial-temporal visual context where the activity occurred.

UGM can also be used to recognize abnormal activities. For example, Hu and Yang presented a probabilistic and goal-correlation based two-level framework to deal with concurrent and interleaving goals from observed activity sequences [23]. At the low level, skip-chain CRF was used to estimate whether a newly goal exist in the given observed activity. While at the high level, relational graph was adopted to represent the correlation between different goals.

### 2.3. Manifolds Geometry

Besides above methods in Euclidean spaces, there are also some emerging and interesting techniques, for example manifolds geometry. In [29], Liu *et al*. used Grassmann manifolds to classify human actions. A tensor was characterized as a point on manifold, and then mapped to the geodesic distance on this manifold. Recently, Harandi *et al*. compared Riemannian manifolds with several state-of-the-art methods to check their performance of representing human activities [30]. They conducted several vision based classification experiments, including gesture recognition and person re-identification. And the experimental results indicate considerable improvements in discrimination accuracy.

In this kind of methods, human activities were related to a particular matrix manifold. Human motion patterns can then be characterized using some transformation. Besides Grassmann and Riemannian manifolds, the matrix manifolds of interest also include Lie groups, and Stiefel manifolds. Lui presented a good description of the recent advance in matrix manifolds for computer vision, and introduced its applications in human activity recognition. For details can refer to [31].

## 3. Systems for Activity Recognition

**Figure 2.**
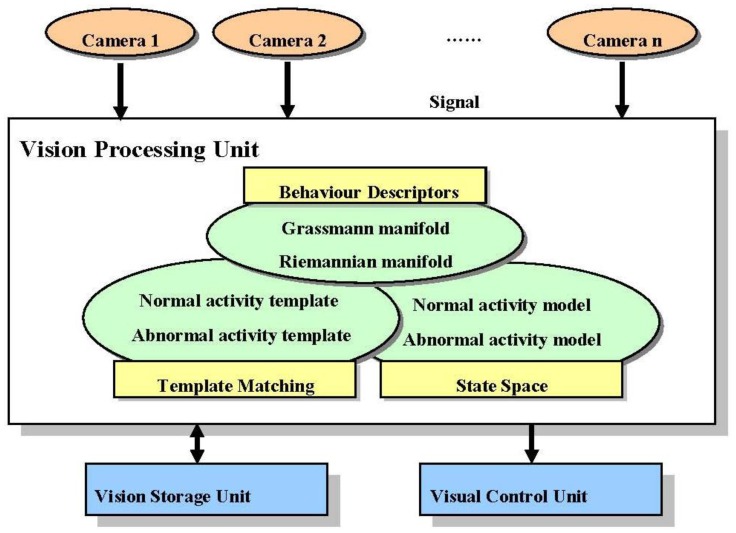
The basic framework of an automatic vision surveillance system.

Vision based surveillance systems can be used to detect, analyze, and recognize activities. In [32–34], good descriptions of vision processing techniques in surveillance systems were presented. As illustrated in Figure 2, the basic framework of an automatic vision surveillance system is composed of a set of cameras, vision processing unit, vision storage unit, and visual control unit. These units were interconnected through a network or other kind of device. In the framework, vision processing unit plays an important role, which contains the key techniques for activity recognition.

In the past, large amount of vision based surveillance systems outfitted with inexpensive cameras were proposed. We will summarize the research projects approved in this domain. Typical systems include: Closed Circuit Television (CCTV) [35], Pfinder [36], W4 [37], Human Identification at a Distance (HID) [38], Context Aware Vision using Image-based Active Recognition (CAVIAR) [39,40], BEHAVE [41], Visual Surveillance and Monitoring (VSAM) [42], Project from the Center for Biometrics and Security Research (CBSR) [43], IBM Smart Surveillance System (S3) [44], *etc*. From the perspective of system architecture and technology, Kumar divided the evolution of vision based surveillance systems into four stages [34]. Table 2 illustrates the past approved research projects on activity recognition in these four stages.

The first generation vision-based surveillance systems consisted of a number of Charge Couple Diode (CCD) cameras, which were connected with a set of monitors using automatic control switches. For example, Nwagboso proposed a CCTV system to assist understanding the events in traffic networks and finally provide better traffic control, incident management and traffic law enforcement [35]. The CCD cameras can continuously trigger image saving routines and monitor accident black spots, thus they can be used as a forensic tool after vehicle crashes have taken place.

However, the widespread deployment of CCD cameras resulted in more expensive and ineffective human supervision. In order to automatically detect alarming events proactively rather than record them passively, second generation surveillance systems were developed. The Pfinder and W4 developed by the MIT Media Laboratory and the University of Maryland in the early years belong to this kind of systems [36, 37]. The significant feature of these systems lies in its ability to provide robust detection, tracking and classification algorithms. Besides Pfinder and W4, several recently emerged second generation surveillance systems exist. For example, the HID project sponsored by the Defense Advanced Research Projection Agency (DARPA) fused biometric technologies into a human identification system to detect, recognize and identify humans at significant standoff distances [38]. The incorporation of biometric technologies can help to enable faster and more accurate identification of humans, and thus can provide useful early warning support for force protection and homeland defense to deal with terrorists, criminals, and other human-based threats. Differently, the CAVIAR project funded by the Information Society Technology (IST) made use of various information including task, scene, function, and object contextual knowledge to provide rich description for local images through hierarchal visual processes [39,40]. The information can enable CAVIAR to perform its function in detecting nighttime crime and classifying customers' commercial behaviors. In order to filter out uninteresting normal activities and not occurring activities from video stream, the UK's Engineering and Physical Science Research Council funded the BEHAVE project undertaken by the University of Edinburgh [41]. BEHAVE, using the dynamic Hidden Markov Model to track individuals, can detect and discriminate between similar interactions. Besides, global probabilistic models were adopted to solve the inconformity during the tracking of individuals in crowd scenes, where images were obtained in a short-time.

In order to achieve wide area surveillance, third generation surveillance systems were designed using distributed, heterogeneous and synergistic cameras. A typical example of this system is the VSAM project supported by DARPA [42]. Cooperative multi-sensors were used in VSAM to track human and vehicles persistently in a cluttered environment. The main goal of VSAM was to monitor the condition in battlefields through automatically collecting real-time information, and assisted improving the situational awareness of commanders and staff. The CBSR at Institute of Automation, Chinese Academy of Sciences developed an intelligent visual surveillance system, which can ensure public safety and enhance protection from terrorist attacks [43]. This system can recognize anomaly and abnormal activities, detect abandoned or removed objects, and track multiple objects at night time; moreover, it also can display overall information in panoramic monitoring screen.

Recently, fourth generation surveillance systems were proposed so as to provide real time event alerts and long term statistical patterns in large scale distributed video surveillance systems. This kind of systems was built on top of existing IP-network infrastructure using wireless networks and networked digital video cameras [34]. For example, IBM Corporation developed a middleware named S3 to provide video based activities analysis capabilities [44]. S3 is a kind of the fourth generation surveillance system. S3 can not only automatically monitor a scene, but also perform surveillance data management, event based retrieval, long term activity pattern statistics, and web based real time events alarm. There are two main components in S3. The first one was Smart Surveillance Engine (SSE), which provided the front end video analysis capabilities; and the other one was Middleware for Large Scale Surveillance (MILS), which enabled data management and retrieval functions. These two components can be used along with the IBM DB2 and IBM WebSphere Application Server to realize a series of functions, such as local and web based real time surveillance and event notification, web based surveillance event retrieval, and web based surveillance event statistics.

## 4. Evaluation Metrics for Activity Recognition

Effectively evaluating the performance of methods and systems for activity recognition in videos or image sequences is important for the improvement of surveillance algorithms in theory, and also for the selection of proper surveillance solutions towards practical applications. Based on past work, much effort has been made towards generating metrics to evaluate the performance of video based automatic surveillance systems. As illustrated in Table 3, we will review some of the recent efforts.

### 4.1. Research Projects for Performance Evaluation

The earliest effort in performance evaluation started with the Video Analysis and Content Extraction (VACE) program in the year 2000. VACE, supported by Advanced Research and Development Activity (ARDA), aimed to develop novel algorithms and implementations to analyze video content including newscasts, meetings, and surveillance [45–47]. Thus, VACE pays special attention to tasks such as detection and tracking of text, faces, person's positions, *etc*. The performance evaluation initiative in VACE is carried out by the University of South Florida (USF) under the guidance of National Institute of Standards and Technology (NIST). The evaluation was based on the framework by Kasturi *et al*. [53], which is a well established protocol for performance evaluation of object detection and tracking in video sequences. Evaluation criterions in VACE vary according to different tasks. For the detection tasks, VACE takes use of the Sequence Frame Detection Accuracy (SFDA) metric to obtain the detection accuracy (misses and false alarms) and the detection precision (spatial alignment); while for the tracking tasks, Average Tracking Accuracy (ATA) metrics is used to measure both tracking accuracy (number of correct trackers) and tracking precision (spatial and temporal accuracy).

The Performance Evaluation of Tracking and Surveillance (PETS) workshop is another endeavor [48]. This yearly workshop investigated moving object detection and tracking in the earliest years; and turned to focus on density estimation, left luggage detection, and activity recognition in recent years. General outdoor surveillance benchmark datasets and online evaluation service were provided in this workshop for the participants to evaluate their systems. Unlike VACE, all metrics in PETS are defined as error measures meaning that the lower the score, the better the performance [52]. Like VACE, the metrics in PETS are also task dependent. For the motion segmentation tasks, PETS generated four metrics at the pixel level including Negative Rate, Misclassification Penalty, Rate of Misclassifications, and Weighted Quality Measure; while in case of the tracking tasks, five criteria are used including Percentage of dataset tracked, Average overlap between bounding boxes, Average overlap between bitmaps, Average chamfer distance using the ground truth object bitmap, and Average chamfer distance using the algorithm generated bitmap.

However, in the early days both VACE and PETS lacked evaluation metrics needed for the tasks of event recognition. The detection of activities is difficult to evaluate because the challenge depends strongly on the events to recognize. For instance, it is much easier to detect an intrusion in a zone of interest than a person opening the door [50].

Aiming to address this problem, NIST sponsored another evaluation project named Text REtrieval Conference Video Retrieval (TRECVid) Evaluation for Event Detection from year 2005. TRECVid is a laboratory-style evaluation intended to promote machine learning technology development for event detection in video surveillance [49]. The video source data was mainly derived from the UK Home Office at the London Gatwick International Airport. TRECVid Evaluation for Event Detection was performed through the comparison of the temporal similarity between the annotated reference event observations and the system-detected event observations. And the result of performance was obtained in the form of MD and FA, which can be used to derive Detection Cost Rate (DCR) model and Detection Error Tradeoff (DET) curves. DCR model is a single error measure, which is simply derived from the linear combination of MD and FA. While DET curves aims to graphically depict the tradeoff of these two error types over a wide range of operational points.

ETISEO was approved to evaluate the performance of event detection tasks by comparing “the number of correctly recognized events with the constraint of time”. It is a project starting in January 2005 and sponsored by French government in order to evaluate vision techniques for video surveillance [50,51]. Unlike the above evaluation methods which stand on the algorithm point of view, ETISEO investigates the relationship between algorithms and video sequences. In other words, EITSEO aims at identifying the suitable scene characteristics for a given algorithm and highlighting algorithm weaknesses for further improvements. Besides event detection, other aspects of video surveillance systems can also be evaluated in this project using various metrics. For instance, the accuracy of the 2D or 3D location of objects and the quality of the object shape can be used as criterions for the detection task; while for the tracking tasks, tracking time, object ID persistence and object ID confusion can be used as criterions. ETISEO displays its evaluation results in the form of Receiver Operating Characteristic (ROC) curve defined as a plot of the true positive rate against the false positive rate.

### 4.2. Collaboration between Different Projects

Besides above mentioned performance evaluation projects, many other programs are also created in the past years such as Computers in the Human Interaction Loop (CHIL) [54], Challenge for Real-Time Event Detection Solutions (CREDS) [55], *etc*. However, the existence of many concurrent metrics makes it difficult to compare them in a fair manner as they are not formalized in the same way [56]. Since most of the performance evaluation programs share the same motivation of developing novel algorithms for detection, tracking, and behavior recognition of humans and objects in video sequences. Technology mapping/transfer among individual projects may contribute to a fair comparison and fast research growth. In addition, current performance evaluation is still limited to short sequences. These sequences and their annotation are often available only to those who created them [51]. It is also necessary to provide benchmark dataset and ground truth data with common evaluation setup to all researchers. Table 4 shows some of the recent collaboration efforts.

The Classification of Events, Activities and Relationships (CLEAR) Evaluation Workshop is the first attempt to bring together two projects: VACE and CHIL [57]. This collaboration has achieved great success. The evaluation metrics provided in CLEAR are widely accepted as an effective and informative assessment of system performance. In addition, CLEAR provides the availability of more data to the research community for algorithm development.

After that, Manohar *et al*. [58] presented a qualitative comparison of detection and tracking tasks in the VACE and the PETS programs. Performance metrics, along with other vital aspect such as the framework, the tasks and ground truth data, are compared thoroughly in this comparison. They believed that the identification of right set of metrics can be achieved through continuing collaboration of the task definitions, database development, *etc*. In 2010, PETS started to evaluate the object detection and tracking tasks based on the SFDA and ATA metrics, which are formally used by the VACE and CLEAR programs [59]. As a result, researchers can evaluate the detection and tracking performance of their systems using the same metrics (SFDA and ATA) and more data (both from CLEAR and PETS).

For the event detection task, Desurmont *et al*. [56] performed mapping the metrics in TRECVid, CREDS and their project. There metrics are compared using a toy example, where events have a temporal duration and are represented as a time interval with a beginning and ending time. Results indicated the metrics in TRECVid project is fully consistent. Based on the problem formalization of TRECVid, the authors further proposed a faster implementation for duration-less events [60].

## 5. Conclusions

In this paper, we present an overview of recent techniques for vision based activity recognition. We have summarized previous work from different technical viewpoints. In addition, we have also reviewed and past approved surveillance systems, as well as the research projects for performance evaluation.

However, there are still some problems that need to be solved in the future. Robust recognition of activities depends on rapid human motion detection, reliable motion tracking, and accurate data analysis [24,33]. These tasks are challenging for several reasons, such as noise and uncertainty backgrounds. Even with robust human motion detection and tracking, activity recognition may still pose great difficulties, including variance in the appearance of particular events, similarity in the appearance of different events, lack of specific background information which may contain large amount of prior knowledge, *etc*.

Besides, the evaluation of the performance of these tasks is another important issue. Although much work has been done on evaluating the performance of activity recognition, standardized evaluation metrics and benchmark datasets are still lacking. For different algorithms and datasets, it is difficult to evaluate and compare their performance with others. Moreover, as far as we know, most of current investigations are focused on the evaluation of algorithms. There is scarcely any evaluation towards the performance of practical surveillance products. Multiple metrics and criterions may help researchers to evaluate their algorithms more effectively. However, it is not convenient for the manufacturer and the end user. A comprehensive metric can be helpful for them to select a suitable surveillance system from large numbers of products. Unfortunately, current evaluation metrics can only reflect part of overall performance; comprehensive criteria are still lacking.

## Figures and Tables

**Table 1.** Methods for human activity recognition.

	**Category**	**Method**		**Articles**
**Activity recognition**	Template matching	Normal activity template		[11–14]
		Abnormal activity template		[15]
	State space	Normal activity model	DGM	[16–18]
			UGM	[19,20]
		Abnormal activity model	DGM	[21,22]
			UGM	[23]

**Table 2.** Past surveillance systems based on activity recognition.

	**Evolution**	**Characteristic**	**Systems**
**Surveillance system**	First generation	Human supervision	CCTV [35]
	Second generation	Automatic real time recognition	Pfinder [36], W4 [37], HID [38], BEHAVE [41], CAVIAR [39,40]
	Third generation	Wide area surveillance	VSAM [42], CBSR [43]
	Fourth generation	Long term activity pattern statistics	IBM S3 [44]

**Table 3.** Past research projects on performance evaluation.

	**Tasks**	**Projects**
**Research projects for performance evaluation**	Detection and tracking	VACE [45–47]
		PETS in the early years [48]
	Event detection	TRECVid [49]
		ETISEO [50,51]
		PETS in recent years [52]

**Table 4.** Past efforts on the collaboration of different projects.

	**Efforts**	**Projects**
**Collaboration projects**	CLEAR [57]	VACE
		CHIL
	Manohar [58]	VACE
		CLEAR
		PETS
	Desurmont [56]	TRECVid
		CREDS

## References

[1] Lacko D. (2011). Motion Capture and Guidance Using Open Source Hardware. Master Thesis.

[2] Kautz H. A. (1987). Formal Theory of Plan Recognition. Ph.D. Thesis.

[3] Sheikh Y., Shah M. (2005). Bayesian modeling of dynamic scenes for object detection. IEEE Trans. Pattern Anal. Mach. Intell..

[4] Yilmaz A., Javed O., Shah M. (2006). Object tracking: A survey. ACM Comput Surv..

[5] Ko T., Shah M. A survey on behavior analysis in video surveillance for homeland security applications.

[6] Lavee G., Rivlin E., Rudzsky M. (2009). Understanding Video Events: A Survey of Methods for Automatic Interpretation of Semantic Occurrences in Video. IEEE Trans. Syst. Man Cybern Part C.

[7] Popoola O.P., Wang K. (2012). Video-Based Abnormal Human Behavior Recognition—A Review. IEEE Trans. Syst. Man Cybern Part C.

[8] Liao L. (2006). Location-Based Activity Recognition. Ph.D. Thesis.

[9] Aggarwal J.K., Ryoo M.S. (2011). Human activity analysis: A review. ACM Comput. Surv..

[10] Casdagli M., Eubank S., Farmer J.D., Gibson J. (1991). State space reconstruction in the presence of noise. Physica D.

[11] Bobick A., Davis J. (2001). The recognition of human movement using temporal templates. IEEE Trans. Pattern Anal. Mach. Intell..

[12] Oren M., Papageorgiou C., Sinha P., Osuna E., Poggio T. Pedestrian detection using wavelet templates.

[13] Ben-Arie J., Wang Z., Pandit P., Rajaram S. (2002). Human activity recognition using multidimensional indexing. IEEE Trans. Pattern Anal. Mach. Intell..

[14] Lu W., Okuma K., Little J. (2009). Tracking and recognizing actions of multiple hockey players using the boosted particle filter. Image Vis. Comput..

[15] Khalid S., Naftel A. Classifying spatiotemporal object trajectories using unsupervised learning of basis function coefficients.

[16] Wilson A.D., Bobick A.F. Recognition and interpretation of parametric gesture.

[17] Duong T., Bui H., Phung D., Venkatesh S. Activity recognition and abnormality detection with the switching hidden semi-Markov model.

[18] Duong T., Phung D., Bui H., Venkatesh S. (2009). Efficient duration and hierarchical modeling for human activity recognition. Artif. Intell..

[19] Chieu H., Lee W., Kaelbling L. (2006). Activity recognition from physiological data using conditional random fields. Tech. Rep. Singapore MIT Alliance Symp..

[20] Yin J., Hu D., Yang Q. Spatio-temporal event detection using dynamic conditional random fields.

[21] Yin J., Meng Y. (2009). Abnormal behavior recognition using self-adaptive hidden markov models. Lect. Notes Comput. Sci..

[22] Loy C.C., Xiang T., Gong S. (2009). Surveillance video behaviour profiling and anomaly detection. Proc. SPIE.

[23] Hu D.H., Yang Q. CIGAR: Concurrent and interleaving goal and activity recognition.

[24] Wang L., Hu W., Tan T. (2003). Recent developments in human motion analysis. Pattern Recognit.

[25] Xiang T., Gong S. (2008). Video behavior profiling for anomaly detection. IEEE Trans. Pattern Anal. Mach. Intell..

[26] Robertson N., Reid I. (2006). A general method for human activity recognition in video. Comput. Vis. Image Underst..

[27] Bishop C.M. (2006). Pattern Recognition and Machine Learning.

[28] Vail D.L., Veloso M., Lafferty J.D. Conditional random fields for activity recognition.

[29] Lui Y., Beveridge J.R., Kirby M. Action classification on product manifolds.

[30] Harandi M.T., Sanderson C., Wiliem A., Lovell B.C. Kernel analysis over riemannian manifolds for visual recognition of actions, pedestrians and textures.

[31] Lui Y. (2012). Advances in Matrix Manifolds for Computer Vision. Image Vision Comput..

[32] Shin J., Kim S., Kang S., Lee S., Paik J., Abidi B., Abidi M. (2005). Optical flow-based real-time object tracking using non-prior training active feature model. Real Time Imaging.

[33] Amer A., Regazzoni C. (2005). Introduction to the special issue on video object processing for surveillance applications. Real Time Imaging.

[34] Kumar P., Mittal A., Kumar P. (2008). Study of robust and intelligent surveillance in visible and multi-modal framework. Informatica.

[35] Nwagboso C. (1998). User focused surveillance systems integration for intelligent transport systems. Advanced Video-based Surveillance Systems.

[36] Wren C.R., Azarbayejani A., Darrell T., Pentland A.P. (1997). Pfinder: Real-time tracking of the human body. IEEE Trans. Pattern Anal. Mach. Intell..

[37] Haritaoglu I., Harwood D., Davis L.S. (2000). W4: Real-time surveillance of people and their activities. IEEE Trans. Pattern Anal. Mach. Intell..

[38] Toole A.J., Harms J., Snow S.L. (2005). A video database of moving faces and people. IEEE Trans. Pattern Anal. Mach. Intell..

[39] List T., Bins J., Fisher R.B., Tweed D., Thorisson K.R. Two approaches to a plug-and-play vision architecture—CAVIAR and psyclone.

[40] Tweed D., Fang W., Fisher R., Bins J., List T. Exploring techniques for behavior recognition via the CAVIAR modular vision framework.

[41] Andrade E.L., Blunsden S., Fisher R.B. Modelling Crowd Scenes for Event Detection.

[42] Collins R.T., Lipton A.J., Kanade T., Fujiyoshi H., Duggins D., Tsin Y., Tolliver D., Enomoto N., Hasegawa O., Burt P. (2000). A System for Video Surveillance and Monitoring: VSAM Final Report.

[43] Wang L., Tan T., Ning H., Hu W. (2004). Fusion of Static and Dynamic Body Biometrics for Gait Recognition. IEEE Trans Circuits Syst. Video Technol..

[44] Tian Y., Brown L., Hampapur A., Lu M., Senior A., Shu C. (2008). IBM smart surveillance system (S3): Event based video surveillance system with an open and extensible framework. Mach. Vis. Appl..

[45] Kasturi R., Goldgof D., Soundararajan P., Manohar V., Boonstra M., Korzhova V. (2005). Performance Evaluation Protocol for Text, Face, Hands, Person and Vehicle Detection & Tracking in Video Analysis and Content Extraction (VACE-II).

[46] Manohar V., Soundararajan P., Raju H., Goldgof D., Kasturi R., Garofolo J. Performance evaluation of object detection and tracking in video.

[47] Raju H., Prasad S., Sharma P. (2006). Annotation Guidelines for Video Analysis and Content Extraction (VACE-II).

[48] Collins R., Zhou X., Teh S.K. An open source tracking testbed and evaluation web site.

[49] Smeaton A.F., Over P., Kraaij W. Evaluation campaigns and TRECVid.

[50] Nghiem A.T., Bremond F., Thonnat M., Valentin V. ETISEO, performance evaluation for video surveillance systems.

[51] Brown L.M., Senior A.W., Tian Y., Connell J., Hampapur A., Shu C., Merkl H., Lu M. Performance evaluation of surveillance systems under varying conditions.

[52] Young D., Ferryman J. PETS metrics: On-line performance evaluation service.

[53] Kasturi R., Goldgof D., Soundararajan P., Manohar V., Garofolo J., Bowers R., Boonstra M., Korzhova V., Zhang J. (2009). Framework for performance evaluation of face, text, and vehicle detection and tracking in video: Data, metrics, and protocol. IEEE Trans. Pattern Anal. Mach. Intell..

[54] Stiefelhagen R., Steusloff H., Waibel A. CHIL: Computers in the human interaction loop.

[55] Ziliani F., Velastin S., Porikli F., Marcenaro L., Kelliher T., Cavallaro A., Bruneaut P. Performance evaluation of event detection solutions: The CREDS experience.

[56] Desurmont X., Carincotte C., Bremond F. Intelligent video systems: A review of performance evaluation metrics that use mapping procedures.

[57] Mostefa D., Moreau N., Choukri K., Potamianos G., Chu S.M., Tyagi A., Casas J.R., Turmo J., Cristoforetti L., Tobia F. (2007). The CHIL audiovisual corpus for lecture and meeting analysis inside smart rooms. Lang. Resour. Eval..

[58] Manohar V., Boonstra M., Korzhova V., Soundararajan P., Goldgof D., Kasturi R. PETS *vs.* VACE evaluation programs: A comparative study.

[59] Ellis A., Ferryman J. PETS2010 and PETS2009 evaluation of results using individual ground truthed single views.

[60] Desurmont X., Sebbe R., Martin F., Machy C., Delaigle J.F. Performance evaluation of frequent events detection systems.

